# ARFI: from basic principles to clinical applications in diffuse chronic disease—a review

**DOI:** 10.1007/s13244-016-0514-5

**Published:** 2016-08-23

**Authors:** Costanza Bruno, Salvatore Minniti, Alessandra Bucci, Roberto Pozzi Mucelli

**Affiliations:** 1 Department of Radiology, Verona University, P.le LA Scuro 10, 37134 Verona, Italy; 2 Technomed, Verona, Italy

**Keywords:** Elasticity imaging techniques, Anisotropy, Fibrosis, Liver cirrhosis, Kidney diseases

## Abstract

**Abstract:**

The many factors influencing the shear wave velocity (SWV) measured with Acoustic Radiation Force Impulse (ARFI) are examined in order to define the most correct examination technique. In particular, attention is given to the information achieved by experimental models, such as phantoms and animal studies. This review targets the clinical applications of ARFI in the evaluation of chronic diffuse disease, especially of liver and kidneys. The contribution of ARFI to the clinical workout of these patients and some possible perspectives are described.

***Teaching Points*:**

• *Stiffness significantly varies among normal and abnormal biological tissues*.

• *In clinical applications physical, geometrical, anatomical and physiological factors influence the SWV*.

• *Elastographic techniques can quantify fibrosis, which is directly related to stiffness*.

• *ARFI can be useful in chronic diffuse disease of liver and kidney*.

## Introduction

Adopted from clinical practice from long ago, palpation examines the mechanical properties of target organs following the general logic shared by all modern imaging methods: it explores tissues detecting the effects determined by an external force. While conventional radiology and computed tomography extract information from the differential attenuation of X-photons due to density and to the mean atomic number in the body volume sampled, the background of palpation-based diagnosis lies in the relationship linking several diseases to the modifications of tissue stiffness that they induce. The principle of palpation has been recently applied to a series of elasticity-based modalities generally defined as “strain-imaging techniques”, in which diagnostic information derives from the response of the explored tissue (closely related to its elastic properties) to forces inducing mechanical modifications. Both quasi-static (based on manual compression) and dynamic strain-imaging techniques have been developed: these latter use as a stimulus rapidly attenuating shear waves resulting from mechanical vibrations. Such vibrations can be applied either outside (like in Transient Elastography: TE) or in “internal” techniques, directly inside the body.

Acoustic Radiation Force Impulse (ARFI) is a recently developed, dynamic, internal technique that superimposes data concerning tissue elasticity to a conventional gray-scale image generated by commercial ultrasound (US) scanners. In this article, the basic technological principles of the ARFI method, with emphasis on the factors that determine the results, are reported, and its current diagnostic role in diffuse disease (especially of liver and kidney) is critically reviewed.

## ARFI imaging

### Physical principles

**Fig. 1:**
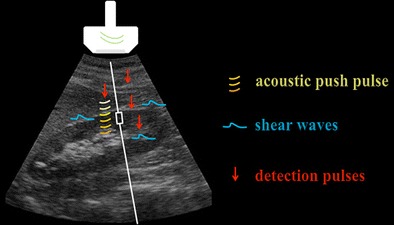
Schematic representation of the ARFI technique. On a conventional gray-scale US image (oblique scan including the right kidney and the lowest portion of the right lobe of the liver), acoustic push pulses (*curved lines*) are generated together with the main US beam. From the push pulses originate shear waves (*dashed horizontal lines*) propagating perpendicular to the main US beam, which are sampled by tracking beams (*arrows*) parallel to the main beam. The actual distance covered by the shear waves before their attenuation is limited: the data concerning tissue response can only be achieved within a small ROI (*rectangular box* along the straight *vertical line*)

In the ARFI technique, the shear waves exciting the target tissue are generated inside a fixed-size (1 x 0.5 cm) region of interest (ROI), placed at the choice of the operator on a conventional gray-scale ultrasound (US) image. In brief, focused, short-duration acoustic push pulses travelling along the main US beam [1] induce within tissues shear stresses, with modalities and intensities depending upon tissue attenuation (mainly due to absorption), acoustic frequency, and intensity of the acoustic beam [2]. In turn, the shear stresses give rise to shear waves that propagate, perpendicular to the main US beam, away from the original region of excitation (Fig. 1). In a theoretical, perfectly homogeneous and isotropic target, the speed of propagation of the shear waves is directly proportional to the density and to the shear modulus of the tissue [1], the latter being related to its elasticity. Therefore, if density does not vary, softer tissues exposed to a given radiation force move farther than stiffer tissues, and, having a lower shear modulus, take longer to reach their peak displacement (on the order of tens of microns), and recover more slowly [3].

Together with the push pulses, low-intensity tracking US beams are continuously emitted parallel to the main beam in order to monitor the tissue displacement. The tracking beams intercept the shear wave front at several predetermined locations and time intervals, which allows a series of data concerning the tissue response to be obtained, such as the time-to-peak displacement and the recovery time (Fig. 1). From these data, mainly through time-of-flight algorithms, quantitative estimates of the speed of propagation of the shear waves, and then of the tissue stiffness, are obtained [1, 4]. Such information can be displayed by ARFI systems as either a map reflecting spatial differences in tissue stiffness, or—more commonly—quantitatively: in most cases, the tissue elasticity is then expressed as shear wave velocity (SWV), usually measured in meters per second (m/s).

### Factors influencing the speed of propagation of the shear waves

**Fig. 2:**
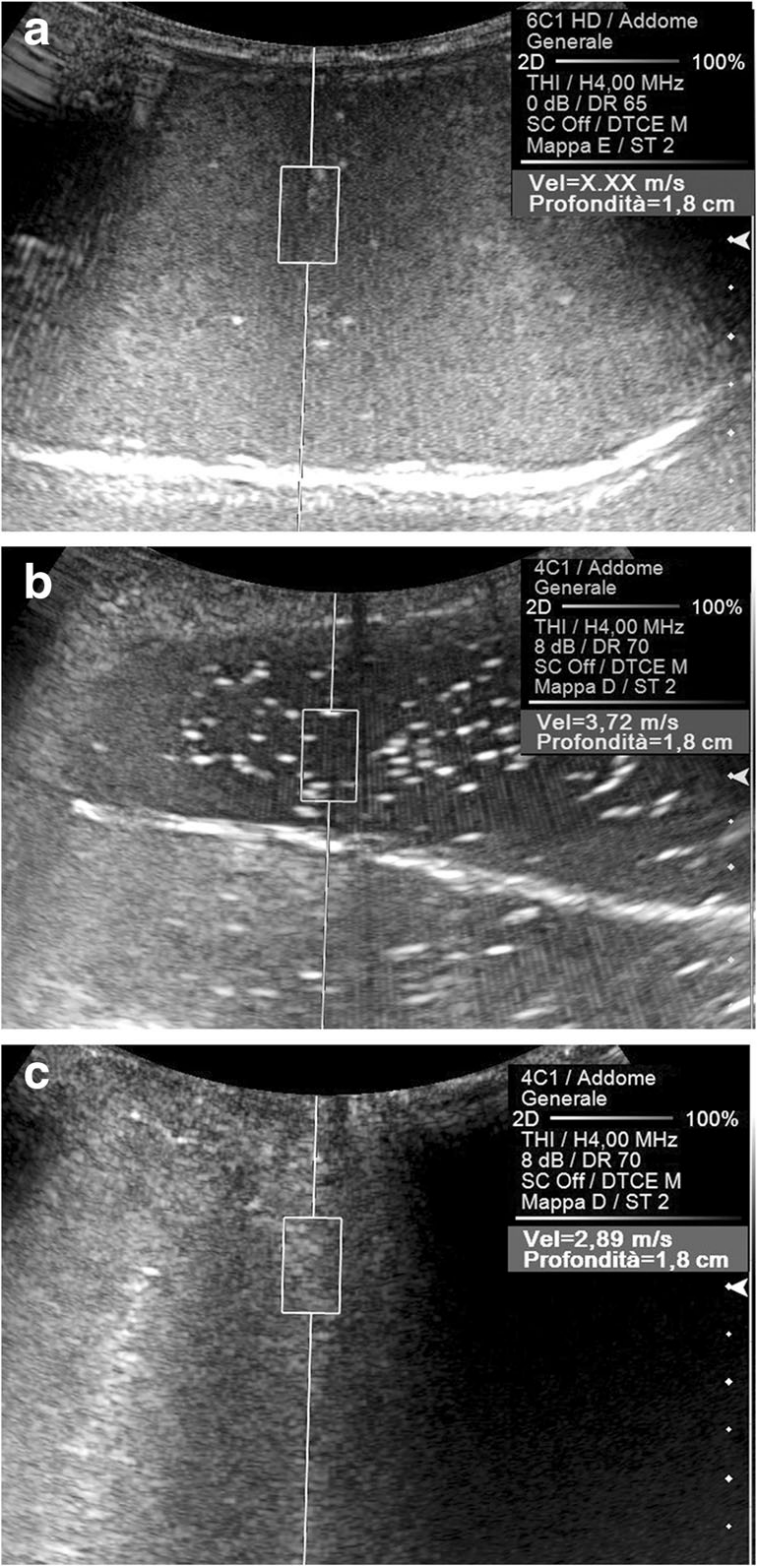
Effect of the elasticity of the target on the speed of propagation of the shear waves. Shear waves do not generate (SWV not measurable: “X.XX”) in pure water (**a**) and travel quite fast (3.72 m/s) in oil (**b**). In a water-oil emulsion (**c**) the SWV (2.89 m/s) is lower than in pure oil

Under ideal experimental conditions (i.e. if both tissue density and all variables related to the US beam and to the generation of shear waves are constant), the elasticity of the target volume is the only determinant of the SWV values measured by ARFI (Fig. 2). In clinical applications, however, the speed at which the shear waves propagate through the medium is strongly influenced by many disturbing factors. The operator must comprehend the physical, geometrical, anatomical and physiological factors potentially capable of modifying the speed of propagation of the shear waves in order to adequately perform the ARFI examination, and thus to avoid dangerous misinterpretation of its results. In the clinical practice, the inappropriate setting of any of the following parameters gives rise to unreliable SWV measurements; moreover, changes of such parameters through follow-up studies may generate variations in the values obtained that could be erroneously attributed to changes in the actual stiffness of the target organs.

**Fig. 3:**
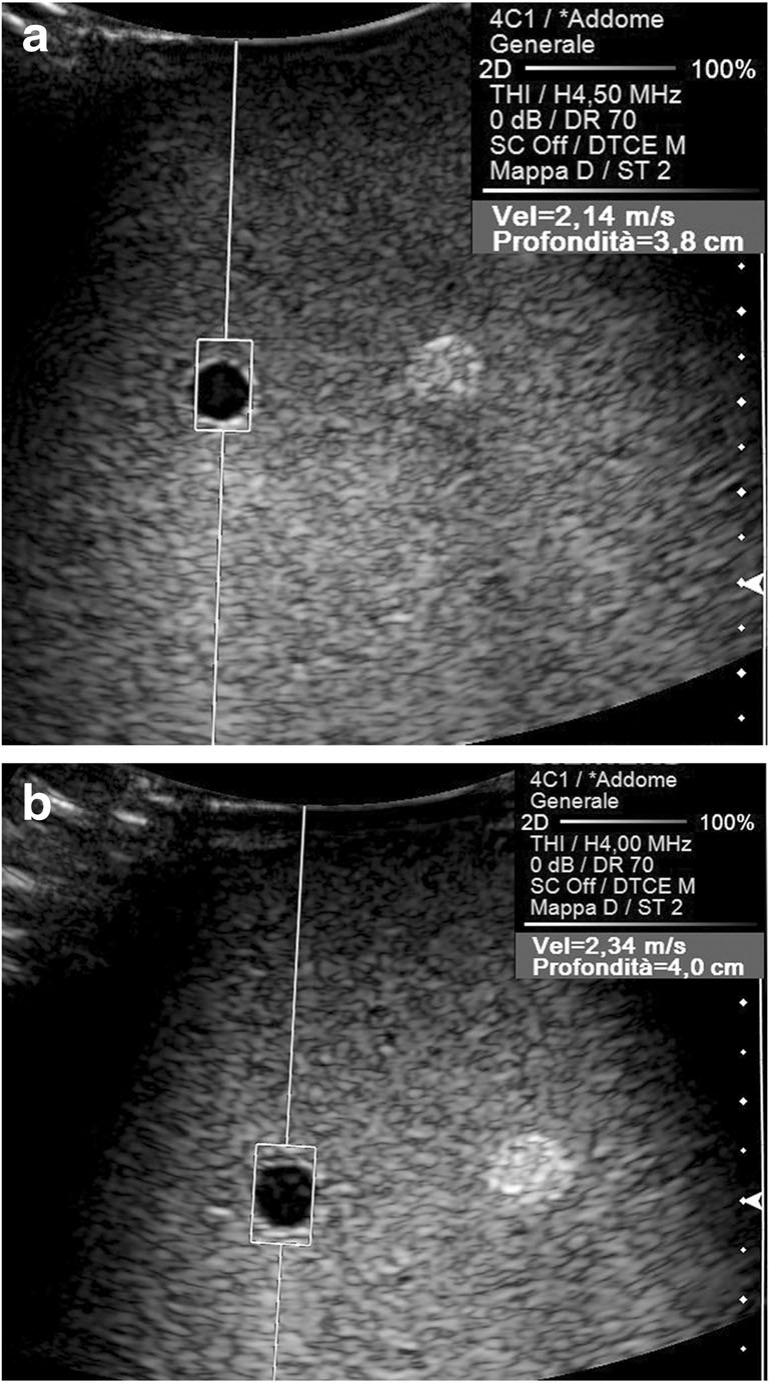
Effect of the wavelength on the speed of propagation of the shear waves. A lower SWV value (2.14 vs. 2.34 m/s) is attributed to a given target (a sphere of gel included into a phantom) using a 4.5 MHz (**a**) compared to a 4.0 MHz (**b**) transmitting frequency
  Among the physical factors extrinsic to the US beam, two variables have to be considered. The stronger the compression manually exerted on the transducer, the higher the tissue density becomes, which increases the speed of propagation of the shear waves (Fig. 4); in an experimental study in which the force applied on the probe was exactly quantified, the SWV values measured in kidney allografts were significantly affected by the degree of compression [6]. In addition, the speed of propagation of the shear waves decreases at greater source-to-target distances, paralleling the progressive attenuation of the pulses generating the shear waves as they travel within tissues (Fig. 5). Significantly lower SWV values were obtained in the deep than in the superficial portion of the right lobe of the liver in healthy volunteers [5, 7], and in the deeper parts of homogeneous phantoms in experimental studies [5, 8]. It is, however, possible that the apparent lower elasticity of deeper targets results from the combined effect of the greater distance from the transducer and of the weaker compression that objects more deeply sited undergo.

- Physical factors affecting the propagation of the shear waves are both intrinsic to the main US beam and extrinsic. The most relevant intrinsic factor is the wavelength: if lower transmitting frequencies are used, lower-frequency pulses are generated, which in turn exert greater acoustic pressure, resulting in faster-travelling shear waves. Exploring both experimental phantoms and normal tissues in vivo with different US frequencies [5], a slight but statistically significant difference in the apparent stiffness was obtained (Fig. 3).

**Fig. 4:**
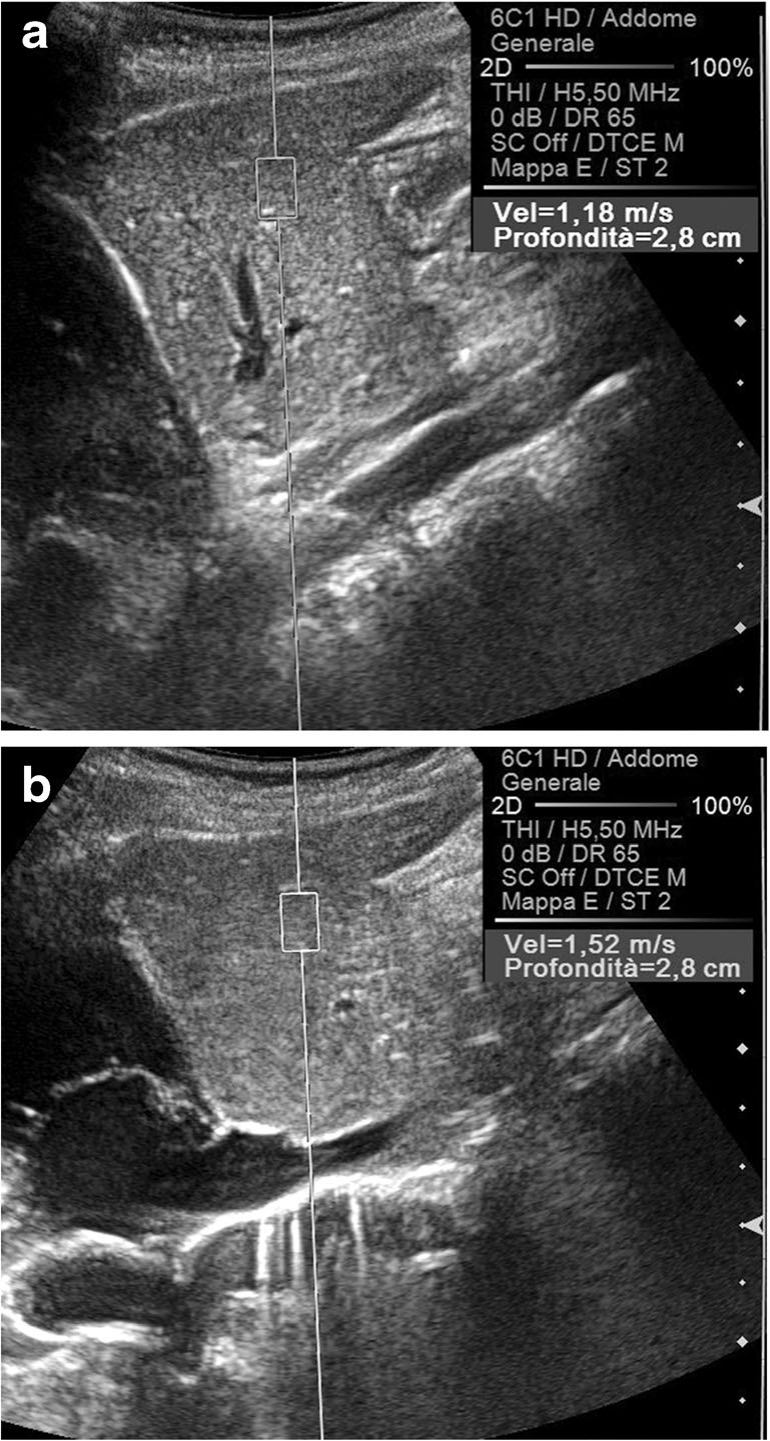
Effect of the compression on the speed of propagation of the shear waves. Longitudinal US scans on the left lobe of the liver in a healthy subject. A lower SWV value (1.18 vs. 1.52 m/s) is measured, exerting on the transducer a mild (**a**) rather than a strong (**b**) manual compression

**Fig. 5:**
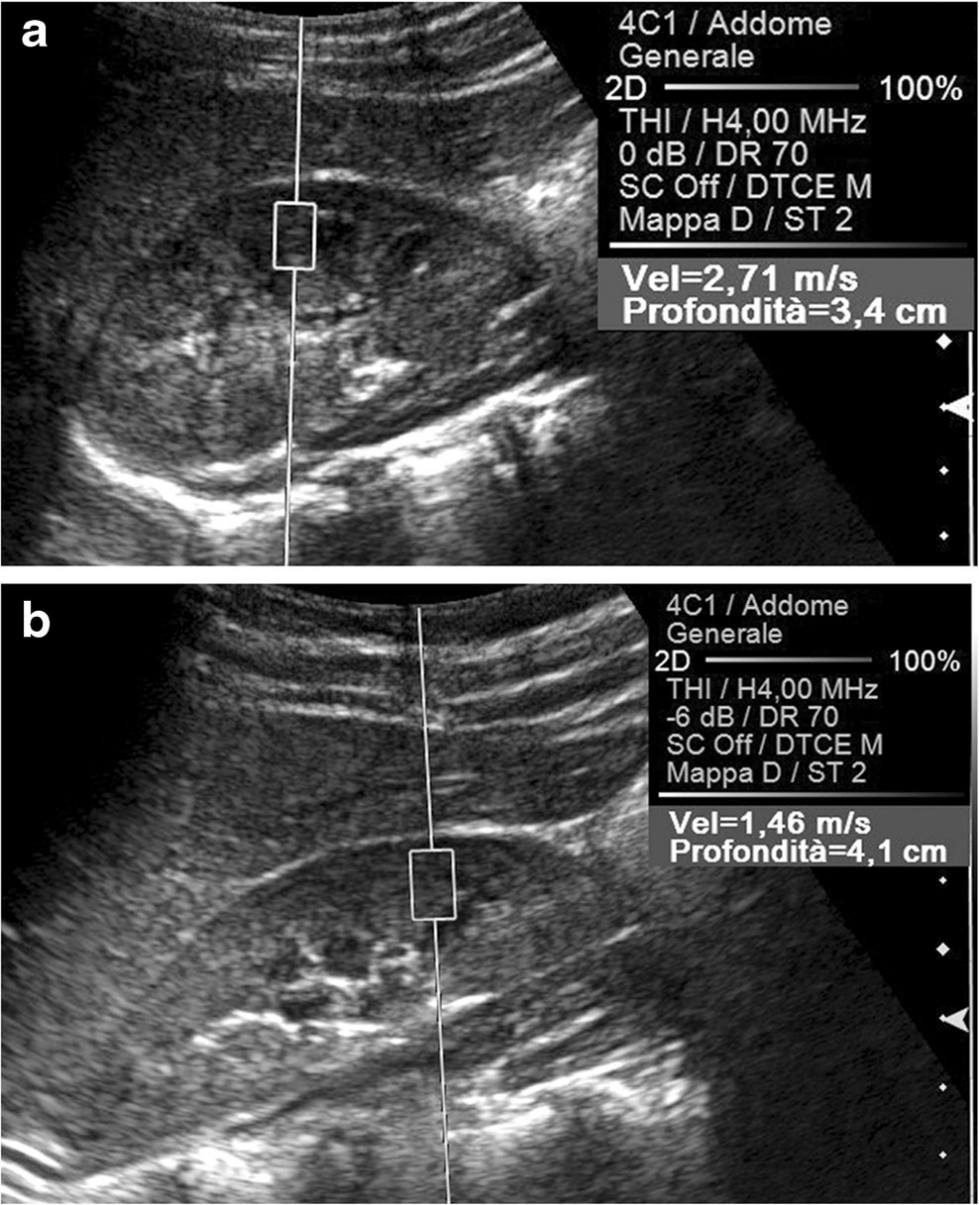
Effect of the source-to-target distance on the speed of propagation of the shear waves. Oblique US scans on the right kidney in a healthy, thin subject. A higher SWV value (2.71 vs. 1.46 m/s) is measured in the renal cortex using a posterior approach (**a**), in which the kidney is closer to the skin surface, compared to using an anterior approach (**b**)

**Fig. 6:**
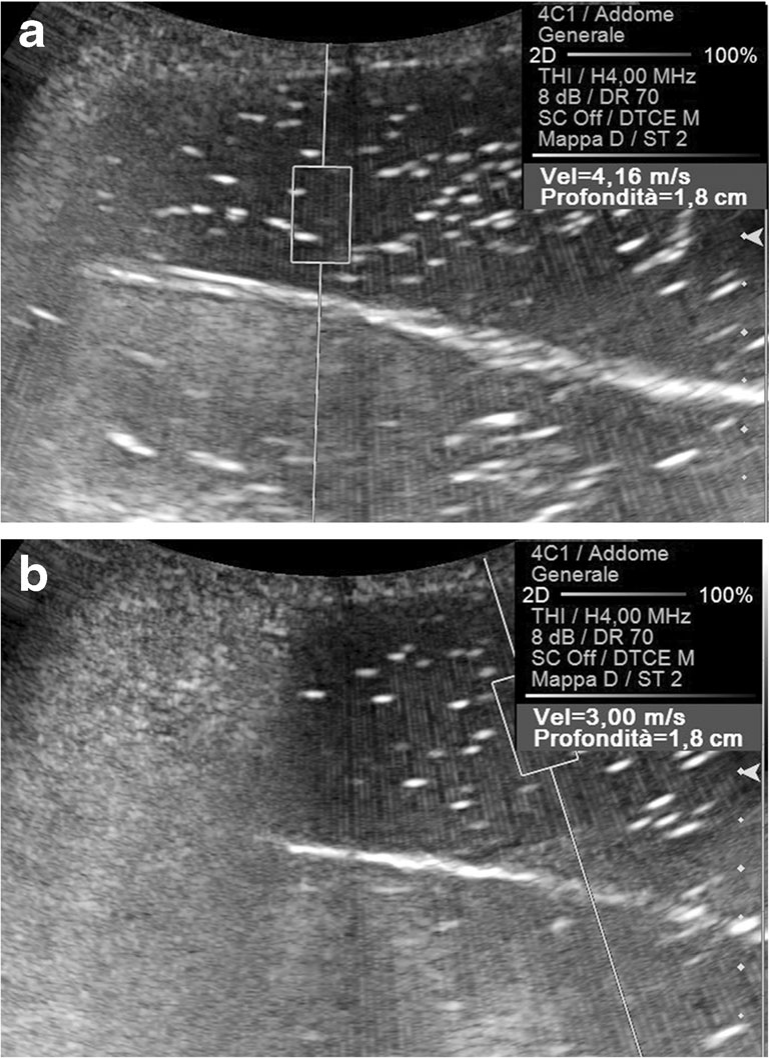
Effect of the orientation of the ROI on the speed of propagation of the shear waves. A higher SWV value (4.16 vs. 3.00 m/s) is attributed to a given target (oil) with a ROI parallel (**a**) rather than oblique (**b**) to the main US beam
- At least two categories of anatomical factors strongly influence the propagation of the shear waves. As a first consideration, within some organs—composed of macroscopic portions each having its own structure—uneven obstacles are opposed to the progression of the shear waves, similarly to the differences in the acoustic impedance regulating the transmission of US beams. Shear waves are expected to meet more architectural disturbances in the sinus than in the cortex of normal kidneys, which is the likely basis of the lower sinusal SWV values (Fig. 7).

- Geometry too plays a role in determining the speed of propagation of the shear waves. The more parallel the ROI is oriented to the main US beam, the higher the SWV results are in a given target (Fig. 6), presumably because of the greater number of interfaces impeding the transmission of the shear waves at increasing obliquities.

**Fig. 7:**
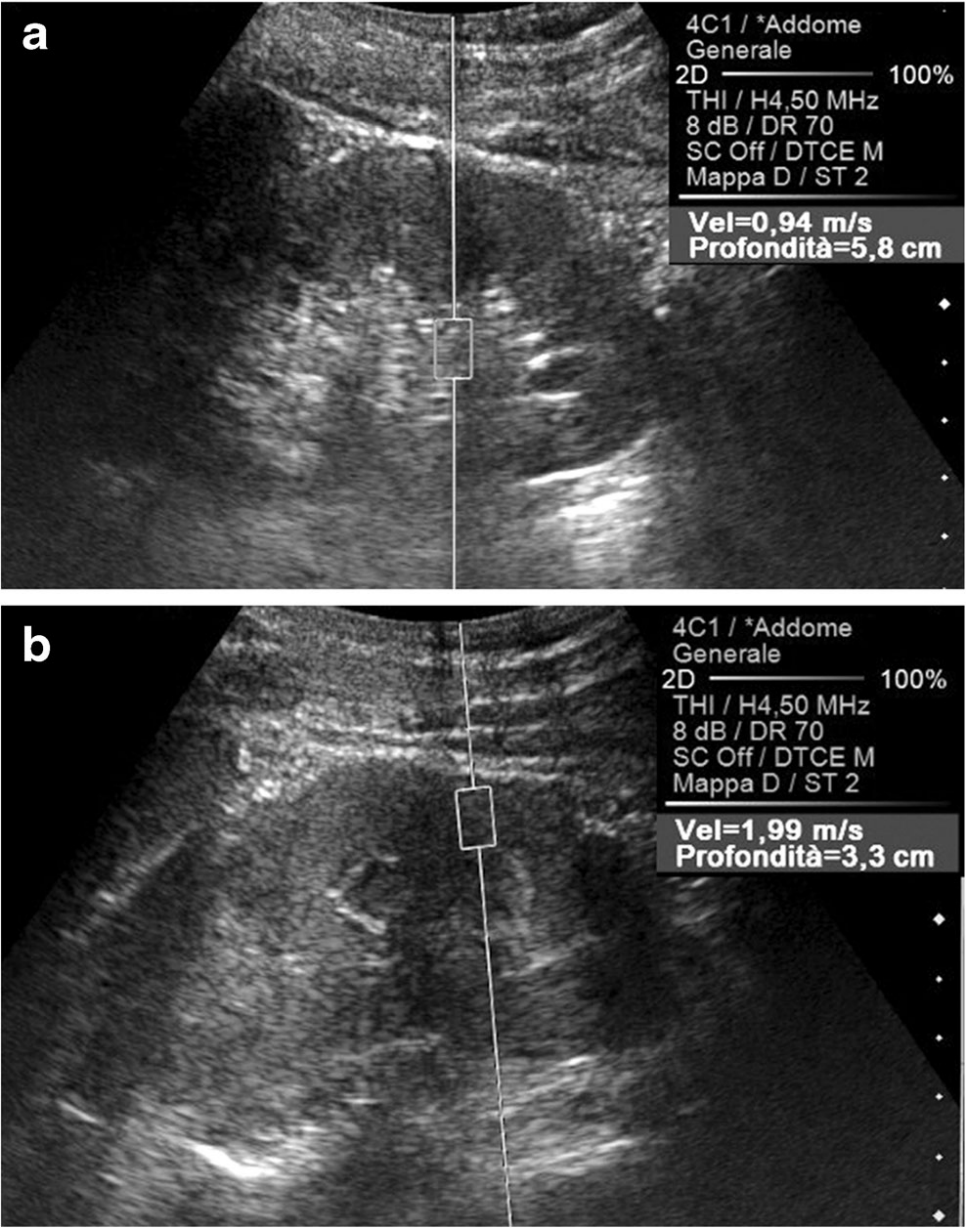
Effect of anatomy on the speed of propagation of the shear waves. Oblique US scans on the right kidney in a healthy subject. A lower SWV value (0.94 vs. 1.99 m/s) is measured in the renal sinus (**a**) compared to in the parenchyma (**b**)
  The most important anatomical factor, however, is anisotropy, which reflects the direction dependence of certain properties that some organs demonstrate (Fig. 8). Most of the current knowledge about the effects of anisotropy on the transmission of shear waves derives from an experimental work by Gennisson on an animal model using supersonic shear waves imaging [9], a variant of the ARFI technique in which the pulses generating the shear waves move more quickly in tissues.

**Fig. 8:**
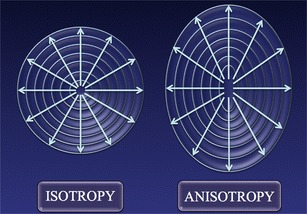
Schematic representation of an isotropic and an anisotropic object: in the latter, properties vary in the different planes. The term comes from ancient Greek words “ἄνισoς” and “τρoπή”
  A marked anisotropy is normally present in the renal medulla, where—within a given renal segment—Henle loops and vasa recta are parallel to each other, all being oriented from the capsule to the papilla (Fig. 9); the same does not happen in the cortex, mostly occupied by glomeruli and by convoluted proximal and distal tubules, with all of these structures approximately spherical in shape. If the main US beam is sent parallel to a renal segment, it generates shear waves travelling perpendicular to the spatially oriented medullary structures, and then encountering multiple interfaces that decrease their speed of propagation, which results in apparently lower elasticity values (Fig. 9). On the contrary, a main US beam perpendicular to Henle loops and vasa recta gives rise to shear waves parallel to such structures and then hindered by much fewer interfaces (Fig. 9), with consequent higher apparent elasticity [9]. In the model of Gennisson, the mean variation of the apparent shear modulus due to the medullary anisotropy was as high as 31.8 % [9], which is consistent with the 40 % fractional anisotropy demonstrated in the renal medulla with diffusion-Magnetic Resonance (MR) [10]. Moreover, a significantly higher speed of shear waves travelling parallel than perpendicular to spatially oriented anatomical structures was observed in muscles [11], in the myocardium [12], and in the brain [13].

**Fig. 9:**
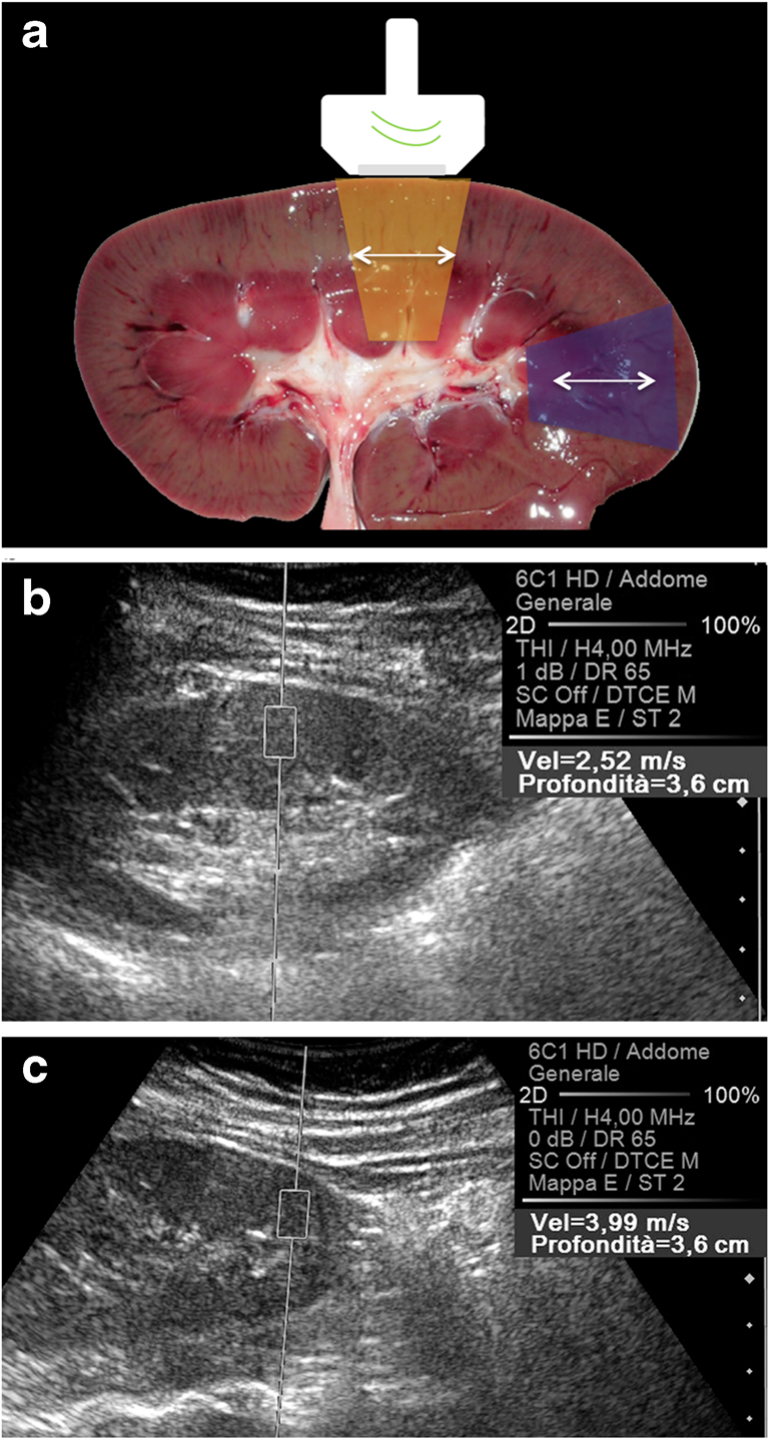
Effect of anisotropy on the speed of propagation of the shear waves. When the axis of the main US beam is parallel to the orientation of the vasa recta and Henle loops (*orange box* in **a**), the shear waves travel perpendicular to these structures and then move slowly (2.52 m/s in **b**). In the opposite condition (*blue box* in **a**), the SWV is higher (3.99 m/s in **c**)
- Like anisotropy, the physiological factors affecting the propagation of the shear waves are especially relevant in the kidneys. In the above-mentioned model [9], a linear relationship of the SWV values to the pressure within the excretory system and to the vascular pressure was demonstrated. In particular, the apparent renal elasticity decreased after ligation of the renal artery (which is in accordance with the results observed with MR-elastography during experimentally induced ischemia) [14], and increased after ligation of the renal vein [9]. This latter behaviour parallels the observations made in otherwise normal organs in which a pressure overload arises as a consequence of an obstacle to the venous outflow, such as in the liver of patients with right heart failure [15] and in the spleen in case of portal hypertension, both in animal models [16] and in vivo [17–19].

## Clinical applications of ARFI

### Safety, feasibility and reproducibility

Prior to the validation of its diagnostic performances, ARFI had to be demonstrated to be a safe technique, quick and easy to perform and providing reproducible results in different settings.

The range of energy of the acoustic beams is similar in common ARFI practice (mechanical index: 1.3 to 1.6) and in conventional gray-scale US. At these levels, the absorption of acoustic energy in biological tissue is expected—besides acoustic radiation force—to generate heat, the amount of which is mainly influenced by transmit frequency and pulse duration. For an individual excitation, the temperature rise is very limited, ranging from 0.02 to 0.2 °C: it takes less energy to displace tissue several microns than to raise its temperature by a fraction of a degree Celsius [1]. Moreover, the thermal safety of ARFI has been experimentally confirmed with both in vitro measurements and finite-element method models [20].

Although radiation forces generated by US beams having intensities and frequencies commonly used in conventional US can cause tissue displacement until a maximum depth shallower than the corresponding B-mode imaging depth [1], from the beginning, ARFI proved to be capable of exploring abdominal targets at a reasonable depth from the skin surface [20], which prompted to its introduction in clinical practice.

The disturbing effect of motion artifacts, caused by both transducer and underlying physiological movements, is easily removed by motion filters; furthermore, multiple reference tracking beams are emitted before push pulses are generated in order to sample baseline motion [1].

As a subsequent historical step in the validation of ARFI technique, images were achieved with a good correspondence to conventional US images, both on cancers after their surgical removal and on normal organs in vivo [20], especially in the abdomen (liver, kidneys, pancreas, spleen), but also in the thyroid and in the testes [21, 22]. However, high standard deviations in the SWV values measured were obtained in these pioneer studies, particularly in the kidneys [21], which limited the enthusiasm for ARFI and inspired several researchers to identify the factors affecting the speed of the propagation of the shear waves. With improving experience of the operators and technical performance of the instruments, however, higher repeatability and reproducibility rates have been achieved, reaching a near-perfect interoperator agreement (intra-class correlation coefficient: 0.99–1.00) in a recent study based on phantoms [8].

With regard to the evaluation of normal and abnormal kidneys, however, a widespread acceptance of the reproducibility of the results of ARFI has not been achieved to date [6, 21, 23–26]. While some authors remain skeptical [6], in the current opinion, ARFI is considered a valuable tool in the exploration of the kidneys, provided that a correct technique is adopted: their anatomical and physiological complexity makes the kidneys more exposed than other organs to most of the factors influencing the propagation of the shear waves. In particular, applying a constant force on the transducer contributes to reducing the variability of the SWV measures, especially in renal allografts, more sensitive than native kidneys to uneven compression because of their more superficial location. A clear identification of the renal segments explored and of their orientation with regard to the US and the shear wave beam is mandatory, due to anisotropy (Fig. 9), for a correct interpretation of the SWV values measured [9, 25]. Urinary obstruction must be ruled out before attributing an increased elasticity to tissue disease [9], and SWV values should be measured soon after micturition [25], especially in transplanted kidneys, where the effect of bladder distension on the pressure in the pyelocaliceal system is magnified by the shortness and the denervation of the ureter [27]. Finally, placing the ROI entirely in the outer renal cortex allows exclusion of the disturbing effects on SWV measurements of both anisotropy (originating in the medulla) and urinary and vascular pressure, the former localized in the calices and the latter in the great medullary vessels [25].

### Evaluation of chronic diffuse disease

#### Liver

Chronic liver disease is very common in clinical practice: its more frequent causes are infection with hepatitis viruses B and C, ethanol abuse, non-alcoholic steatohepatitis, autoimmune hepatitis, and primary biliary cirrhosis. In these patients, a precise estimation of liver fibrosis is crucial both for the planning of treatment (especially in viral hepatitis) and for the assessment of outcome, the degree of fibrosis representing the strongest prognostic indicator [28–30]. On a five-point scale, treatment is commonly considered necessary for levels of fibrosis of 2 (“significant”) or above; grade-3 fibrosis is “severe” and cirrhosis corresponds to grade 4, while F = 0 and F = 1 respectively indicate an absent and a “mild” fibrosis. Liver biopsy is still considered the gold standard in the evaluation of fibrosis [28, 31]; it can also reveal fatty infiltration or specific markers for some diseases, such as Mallory bodies in alcoholic steatohepatitis [30]. However, the average bioptic specimen only represents 1/50,000 of the total liver volume, its dimension and content of portal tracts widely varying, which may constitute a major diagnostic limitation considering the uneven distribution of fibrosis throughout the liver [30]. Moreover, liver biopsy is an invasive procedure: in up to 6 % of cases, complications occur [32], which in 0.04–0.11 % may be life-threatening [30].

On the assumption that a direct proportionality exists between the degree of fibrosis and liver stiffness, both quasi-static and dynamic shear-wave elastographic techniques have been widely used, aimed at a quick, non-invasive and reliable quantification of fibrosis. Among the latter group, TE was introduced in clinical practice more than a decade ago. Several meta-analyses assessed that the measurement of liver stiffness by means of TE, in patients with chronic hepatopathies, significantly correlates with the histological degree of fibrosis [33–35], allowing to reliably identify the stages F ≥ 2.

For historical reasons, therefore, this clinical application of ARFI has been initially validated through a comparison with the already known diagnostic performances of TE, and in no correlative study were significant differences demonstrated between the accuracies of ARFI and TE [33, 36–42]. ARFI, however, had a significantly lower rate of unsuccessful measurements than did TE [38, 43]: unlike TE, ARFI takes advantage of a conventional US image to choose the positioning of the ROI in both planes, and is therefore less sensitive to the presence of ascites and to obesity. Moreover, ARFI performed better than a scoring system based on the visual assessment of conventional US images by experienced radiologists in correlation with Child-Pugh scores and liver function tests, and better than aspartate-to-platelet ratio in predicting severe fibrosis and cirrhosis in patients with alcoholic liver disease [44]. In experimental fibrosis induced in a rat liver model, ARFI proved very reliable in the staging of fibrosis [45].

**Fig. 10:**
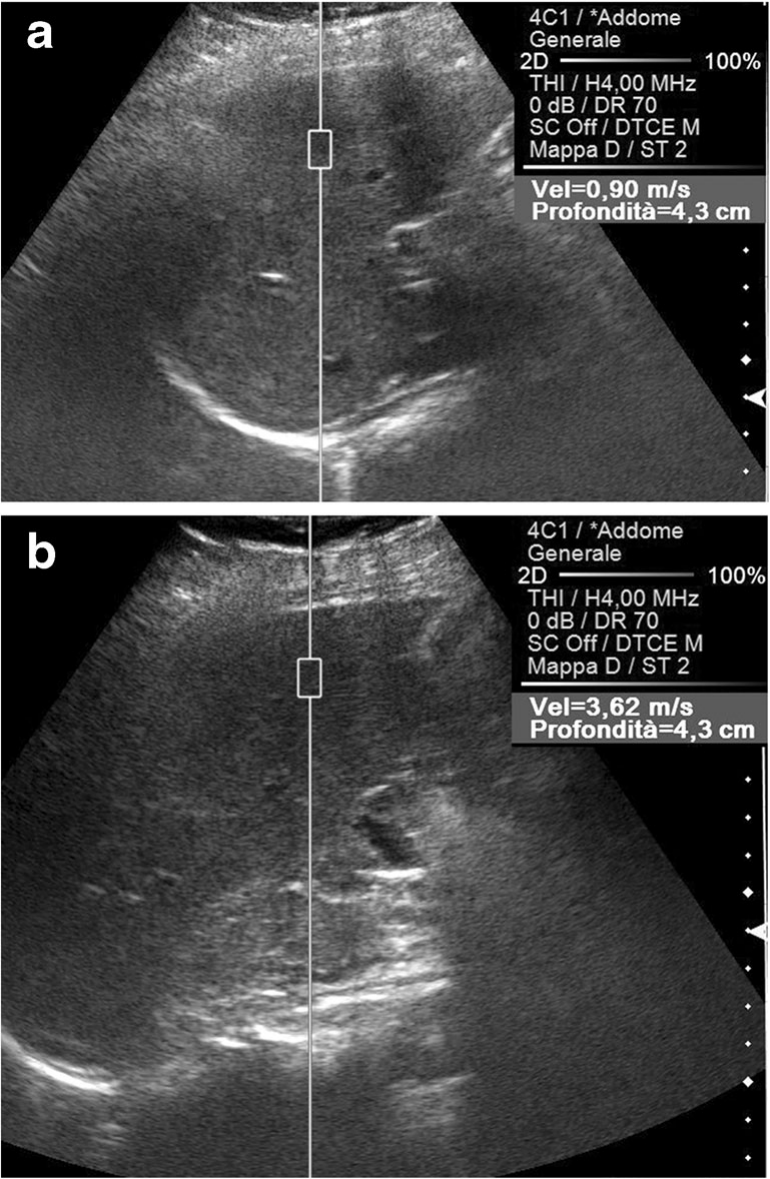
ARFI evaluation of liver. Transversal US scans on the right lobe of the liver in two different patients with chronic liver disease due to hepatitis virus C. The SWV value is lower (0.90 vs. 3.62 m/s) in a 41-year-old man with fibrosis stage 1 at histology (**a**) than in a 51-year-old man with fibrosis stage 3 (**b**)

For the assessment of liver fibrosis (Fig. 10), ARFI scanning is usually performed in the supine position, applying minimal scanning pressure, while the patient is asked to stop breathing [30]; no significant variations were, however, demonstrated measuring the SWV values with or without deep inspiration [7, 15]. An intercostal approach in the right liver lobe is preferred, since in the left lobe, cardiac pulsation causes excessive tissue motion, potentially disrupting shear waves; in addition, measurements in the left lobe tend to be taken closer to the hepatic capsule, where tissue is often more fibrous than in deeper portions [46, 47]. The mean body mass index is known to interfere with the reliability of the procedure [48–51], while equivocal data have been reported concerning possible effects on the SWV values of patient’s age and gender, food intake, patient’s position, and shape of the transducer (convex versus linear probe [5, 7, 15, 49, 50, 52]. Moreover, steatosis and acute inflammation (both likely to occur in chronic liver disease) may interfere with hepatic stiffness [51, 53–55]. The best cutoff points for SWV values emerging from the meta-analyses are 1.31 m/s for the fibrosis stages ≥2 and 1.8 for F = 4 [38]; according to some authors [44, 54], these figures should be corrected according to serum alanine aminotransferase levels. As for its diagnostic performances, many published data report good accuracy of ARFI in chronic viral and alcoholic liver disease [33, 36–39, 48]. In the diagnosis of F ≥ 2, ARFI showed 74 % summary sensitivity and 83 % summary specificity, both reaching 87 % in the diagnosis of F = 4 [38]; the area under receiver operating curve (AUROC) values were 0.84 for F ≥ 2, 0.89 for F ≥ 3 and 0.91 for F = 4 [48]. In a retrospective multicenter study, the correlation between SWV values and histological fibrosis was highly significant (*p* < 0.0001), even if a large overlap of values occurred in the stages of 2 or below, with only F ≥ 2 and cirrhosis being excluded with great certainty [39]. Better results were obtained in European than in Asian patients [39, 56], and in patients infected with hepatitis virus C than in those with virus B [56–58].

As for chronic liver disease due to less common etiologies, ARFI proved reliable in differentiating significant from non-significant steatosis in children with biliary atresia [59] and in adult patients with autoimmune hepatitis [60] and with primary biliary cirrhosis [61]. In patients with non-alcoholic fatty liver disease, in whom an increased risk of developing hepatocellular carcinoma exists only if advanced fibrosis is present, a meta-analysis reported summary sensitivity of 80.2 % and specificity of 85.2 % in the ARFI-based detection of significant fibrosis [62]. Moreover, a reliable differentiation between significant and non-significant fibrosis was obtained in liver allografts in patients infected with hepatitis virus C [63].

**Fig. 11:**
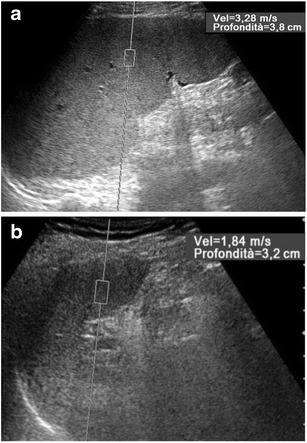
ARFI evaluation of the spleen in patients with alcoholic liver cirrhosis. Longitudinal US scans. The SWV value is lower (1.84 vs. 3.28 m/s) in a 54-year-old man with Child-Pugh score A (**a**) than in a 51-year-old man with Child-Pugh score C (**b**), with severe portal hypertension and recurrent variceal bleeding

The accuracy of ARFI in the prediction of complications of chronic liver disease is more controversial. Although in most cases the risk of bleeding from esophageal varices is low, the current guidelines recommend endoscopy in all patients, in order to identify those who would benefit from prophylactic treatment [64], which led some authors to try a non-invasive measurement of the portal vein pressure. An elevation of liver and spleen elasticity was demonstrated with MR elastography in an animal model of portal hypertension, preceding the development of liver fibrosis [16]. In patients with chronic liver disease, a correlation between liver SWV values and portal hypertension was found by some authors [65, 66] and denied by others [17, 30]. In some studies, the absolute spleen stiffness [17, 18] or its ratio to liver stiffness [19] correlated well with the portal vein pressure (Fig. 11). Hepatic SWV values resulted in significantly higher decompensated liver cirrhosis, whereas no significant relationship was demonstrated between hepatic SWV and the risk of occurrence of hepatocellular carcinoma [30].

#### Kidney

An extension of the excellent results obtained by ARFI in the liver to the evaluation of kidneys could be foreseen on the basis that a hypercellular, high-grade interstitial fibrosis leading to a progressive loss of functioning nephrons is the common final histological pathway of chronic kidney disease, whatever its cause [67], and also developments in renal allograft dysfunction. The utility for a timely diagnosis of chronic kidney disease of both morphological US parameters (such as renal size, cortical thickness, and pelvis diameter) and of the arterial resistive index measured with Doppler US is doubtful [24, 68], which makes renal biopsy often necessary. Moreover, scintigraphy with dimercaptosuccinic acid labelled with technetium-99 m (^99m^Tc-DMSA), a procedure associated with a certain amount of radiation exposure, is recommended by the current guidelines in the evaluation of several pediatric forms, especially in European countries [67, 69]. A pilot study [70] showed that renal parenchymal stiffness measured with TE reflected interstitial fibrosis in allografts, and a good correlation of the cortical stiffness to the global histological deterioration of transplanted kidneys emerged in a study in which supersonic shear wave imaging was used [71].

However, uneven results have been reported concerning the reliability of ARFI in the evaluation of renal allografts. According to Syversveen [6], ARFI can not detect allograft fibrosis, while in another study, a significant increase in SWV values was observed in renal transplants with acute rejection, although not in other pathologies [24]. A significant correlation of SWV values with the estimated glomerular filtration rate of transplanted kidneys was observed by a different group, with 72 % sensitivity and 86.5 % specificity in the diagnosis of allograft dysfunction [72]. In a study in which kidney allografts examined with ARFI underwent biopsy, SWV values did not correlate with interstitial fibrosis and tubular atrophy. However, renal stiffness increased with time after transplantation, especially in the cases of a low kidney weight to body weight ratio, in which a greater amount of arterial blood is conveyed through glomeruli [73]. This result is consistent with Brenner’s hypothesis that hyperfiltration that anatomically or functionally single kidneys undergo (i.e. in patients with unilateral renal agenesis, transplantation, or severe impairment of one kidney) causes damage with subsequent sclerotic changes [74].

In chronic kidney disease of the adult, one author [75] did not obtain a significant relationship of SWV values to the stage of disease and to biochemical indicators of fibrosis. One group reported a direct correlation of renal stiffness to the histological degree of fibrosis, yielding 86 % sensitivity and 83 % specificity with a cutoff value of 1.67 m/s [76]. In some studies, on the other hand, SWV values decreased concurrently with a decline in the estimated glomerular filtration rate [77, 78] and with the progression of histological damage. These results led to the hypothesis [78] that in severe chronic kidney disease, the diminution of renal blood flow due to nephroangiosclerosis may affect the SWV values more than tissue fibrosis does: this might be in accordance with the decrease in kidney elasticity measured with supersonic shear wave imaging in the animal model of Gennisson after ligation of the renal artery [9].

**Fig. 12:**
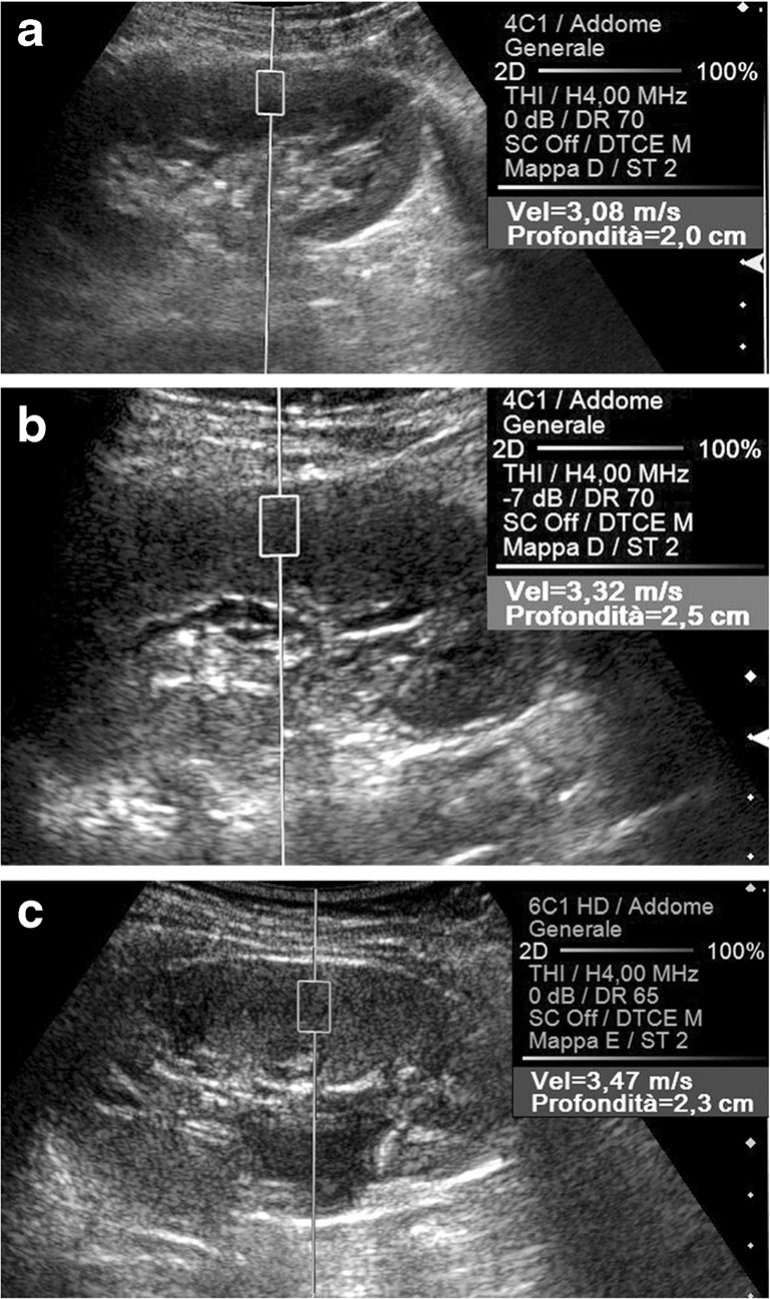
ARFI evaluation of kidney. Oblique US scans on the left kidney of three different 12-year-old boys. The cortical SWV value is lower (3.08 vs. 3.47 m/s) in a healthy control (**a**) than in a boy with chronic kidney disease due to vesicoureteral reflux (**c**) treated at younger age. Intermediate SWV values (3.32 m/s) are measured in the apparently normal left kidney (**b**) of a boy with chronic contralateral disease

As for the pediatric population, in one study in which children younger than 2 years with pelvis dilation were examined, SWV values were significantly higher than in normal controls only when high-grade hydronephrosis was present [79]. In our experience [25], ARFI values were significantly higher than normal in children aged 8–16 years with persistent chronic kidney disease caused by a vesicoureteral reflux successfully treated at early age. The highest SWV values were found in patients whose reflux had been secondary to obstruction, in whom both retrograde urinary flow and abnormal intrarenal pressure had contributed to the initial renal damage. Moreover, we observed significantly higher stiffness in the seemingly normal kidneys contralateral to the affected kidney in children with unilateral disease than in the absolutely normal kidneys of healthy subjects (Fig. 12). The abnormal SWV values measured in kidneys contralateral to the affected ones may reflect the presence of low-grade fibrosis, which is likely due to hyperfiltration damage in functionally single kidneys [74].

#### Pancreas

In acute pancreatitis, SWV values are reported to be significantly higher than in normal pancreas. One group obtained 97 % sensitivity and 93 % specificity in differentiating patients with acute pancreatitis from both normal subjects and patients with chronic pancreatitis using a 2.2 m/s SWV cutoff value [80]; in another study, with 1.63 m/s cutoff, 100 % sensitivity and 98 % specificity were achieved [81]. In both papers, ARFI allowed identification of focal acute pancreatitis; moreover, a progressive SWV decrease in follow-up exams has been associated with recovery, whereas focal zones of low SWV values during the acute phase reflected areas of necrosis [80].

**Fig. 13:**
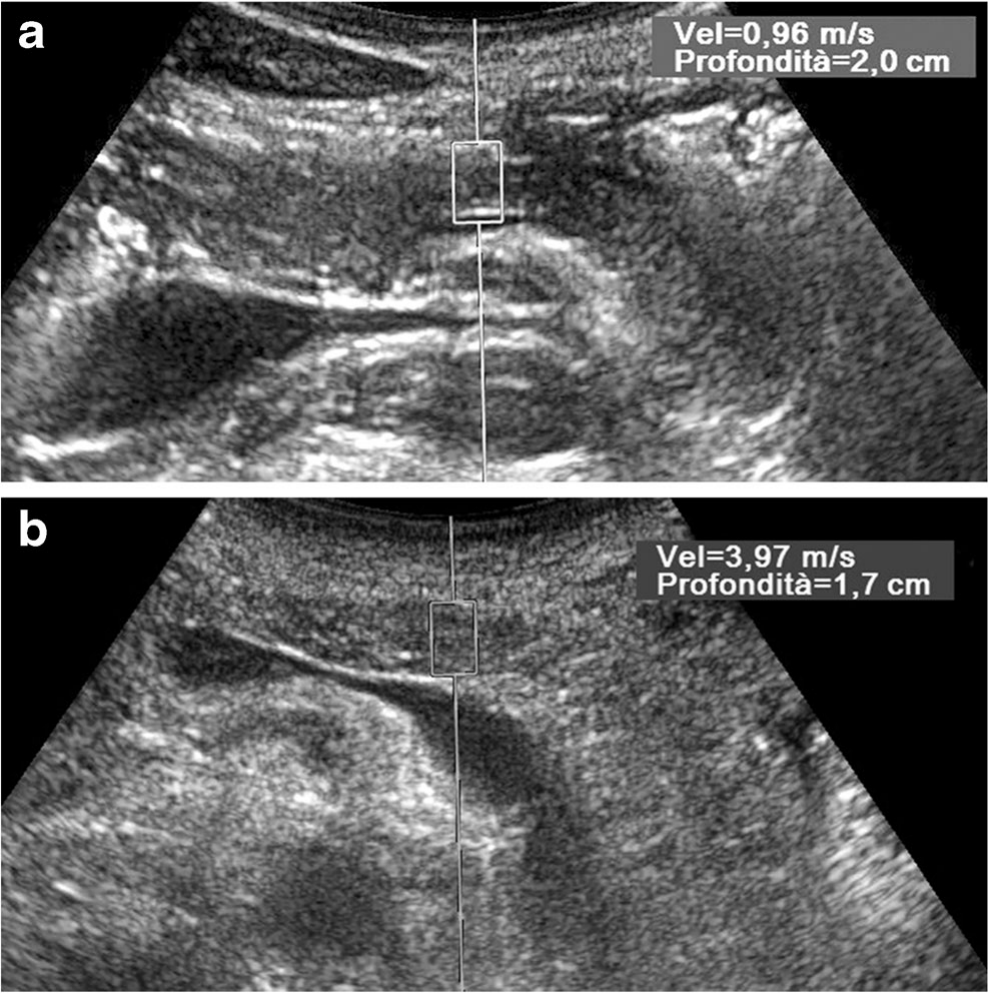
ARFI evaluation of the pancreas. Axial US scans with ROI placed in the pancreatic body. The SWV value is much lower (0.96 vs. 3.97 m/s) in a healthy 38-year-old man (**a**) than in a 48-year-old man (**b**) with severe chronic alcoholic non-obstructive pancreatitis

In one study, SWV values were significantly higher in patients with chronic pancreatitis than in healthy volunteers (Fig. 13): the sensitivity, specificity, PPV, and NPV were 75, 72, 69, and 78 %, respectively [82]. However, no significant SWV differences were detected between normal subjects and patients with chronic pancreatitis in one paper concerning acute pancreatitis [80].

Few other studies regarding the ARFI evaluation of diffuse pancreatic disease can be found. In patients with cystic fibrosis, SWV values were lower when pancreatic insufficiency existed [83]. A higher incidence of post-operative pancreatic fibrosis was demonstrated in patients whose SWV values before pancreatic resection were lower [84].

#### Thyroid

When the stiffness of surgical thyroid specimens was measured, a significant direct relationship of SWV values to the histological degree of fibrosis (regardless of the underlying disease) emerged [85]. As a consequence, in normal thyroids lower SWV values (mean: 1.63 to 2.00 m/s) were observed than in chronic autoimmune thyroiditis (2.43–2.56), yielding 62–77 % sensitivity and 71–79 % specificity [86, 87]. A similar difference was demonstrated between normal thyroids and Basedow-Graves’ disease [87].

## Conclusions

ARFI-based quantification of tissue stiffness is a non-invasive, cheap, safe and quick imaging tool potentially capable of improving the accuracy of US examinations. ARFI reliably quantifies the degree of fibrosis in chronic liver disease, contributing to treatment planning and prognosis assessment, and can probably help in the staging of diffuse chronic disease of other organs, especially the kidneys. Given the high number of factors significantly influencing the measurements, the importance of a rigorous, target-tailored examination technique is critical in order to obtain reliable results.
